# Novel Characterization of Antioxidant Enzyme, 3-Mercaptopyruvate Sulfurtransferase-Knockout Mice: Overexpression of the Evolutionarily-Related Enzyme Rhodanese

**DOI:** 10.3390/antiox8050116

**Published:** 2019-05-01

**Authors:** Noriyuki Nagahara, Mio Tanaka, Yukichi Tanaka, Takaaki Ito

**Affiliations:** 1 Isotope Research Laboratory, Nippon Medical School, Tokyo 113-8602, Japan; 2 Department of Pathology, Kanagawa Children’s Medical Center, Yokohama 232-8555, Japan; mio@zc4.so-net.ne.jp (M.T.); ytanaka@kcmc.jp (Y.T.); 3 Department of Pathology and Experimental Medicine, Graduate School of Medical Science, Kumamoto University, Kumamoto 860-8556, Japan; takaito@kumamoto-u.ac.jp

**Keywords:** knockout mouse, mercaptopyruvate sulfurtransferase, rhodanese, real-time quantitative polymerase chain reaction

## Abstract

The antioxidant enzyme, 3-mercaptopyruvate sulfurtransferase (MST, EC 2.8.1.2) is localized in the cytosol and mitochondria, while the evolutionarily-related enzyme, rhodanese (thiosulfate sulfurtransferase, TST, EC 2.8.1.1) is localized in the mitochondria. Recently, both enzymes have been shown to produce hydrogen sulfide and polysulfide. Subcellular fractionation of liver mitochondria revealed that the TST activity ratio of MST-knockout (KO)/wild-type mice was approximately 2.5; MST activity was detected only in wild-type mice, as expected. The ratio of TST mRNA expression of KO/wild-type mice, as measured by real-time quantitative polymerase chain reaction analysis, was approximately 3.3. It is concluded that TST is overexpressed in MST-KO mice.

## 1. Introduction

**Figure 1:**
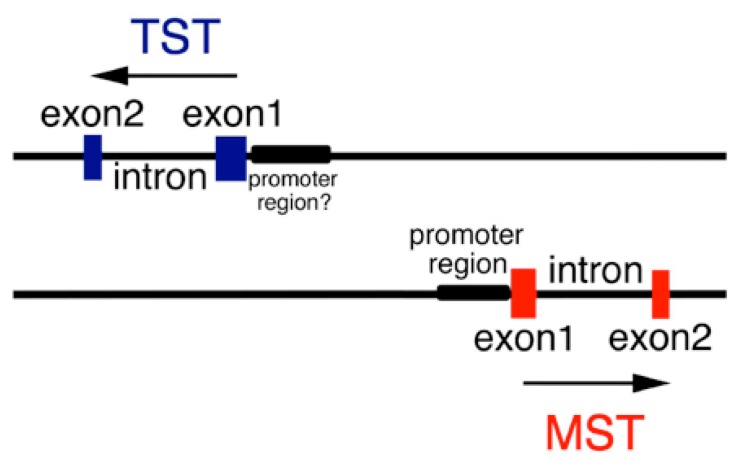
Alignment of TST and MST genes on mouse chromosome 15. Each of two family genes contains two exons and an intron (NC_000081.6). The 5′-Flanking region of the TST gene has not been identified, however, a prompter region exists. Arrows represent transcriptional direction. Details are described in the text.

Rhodanese (thiosulfate sulfurtransferase, TST) and 3-Mercaptopyruvate sulfurtransferase (MST, EC 2.8.1.2) and are ubiquitous, evolutionarily related enzymes distributed in both prokaryotes and eukaryotes [1,2]. Mouse MST gene aligns with TST one on chromosome 15 (Figure 1) (NC_000081.6). The former enzyme is distributed in the cytosol and mitochondria [3] and the latter only in the mitochondria [4]. The 5′ flanking promoter region of MST represents a classical housekeeping gene [5]. On the other hand, the 5′ flanking promoter region of TST has not been clarified. It was reported that human congenital MST deficiency causes mercaptolactate-cysteine disulfiduria (MCDU) [6,7]. Most cases were complicated by mental retardation [8,9], however, in some cases, patients were mentally normal [10,11].

MST performs antioxidative function [12,13,14,15,16,17]. It has been also reported that hydrogen sulfide and polysulfide were produced by MST [18,19,20,21,22] and TST [19,23]. Although these products reportedly have important physiological roles [24,25,26,27], MCDU pathogenesis has not been clarified. To elucidate the underlying pathogenesis, we produced MST-knockout (KO) mice [15]. The gene targeting ranged between 1 and 3606 bp from the initiation codon ATG of mouse MST gene, which covered the exon 1, intron, and partial exon 2 regions. The mice exhibited significant anxiety-like behaviors [15], however the result could not be reconfirmed, which resembled clinical features. To elucidate the etiology, we measured MST and TST activities in KO mice using mitochondrial fractions because rhodanese was distributed only in mitochondria [3]. We also performed real-time quantitative polymerase chain reaction (PCR) with SYBR green I [28], which was useful in measuring the transcription of mRNA. We found that TST was overexpressed and could compensate for the effect of the MST defect in the KO mice, but the mechanism has not been clarified.

## 2. Materials and Methods

### 2.1. Ethics Statement for Animal Experiments

The study was conducted in accordance with the Declaration of Helsinki, and the all experiments were performed in accordance with the guidelines and regulations for the Care and Use of Laboratory Animals, Nippon Medical School (#28-008). Littermate wild-type and KO mice [15] were deeply anesthetized by intra-abdominal pentobarbital injection (100 mg/100 g body weight), and sacrificed by exsanguination.

### 2.2. Subcellular Fractionation of Mitochondria and Cytosol

Livers were excised from three littermate wild-type (#1–3) and three KO mice (#1–3) sacrificed according to the method described above. Mitochondrial and cytosolic fractionations were prepared using a Mitochondria Isolation Kit for Tissue (Thermo Fisher Scientific, Waltham, MA, USA). After an approximately 100 mg portion of each liver was washed with 4 mL of 20 mM potassium phosphate buffer (pH 7.2 at 4 °C) twice, it was cut into small pieces in 800 µL of the same buffer. Each homogenate was prepared using a Potter homogenizer (AR BROWN Co. Ltd., Tokyo. Japan) with a loose-fitted pestle with three strokes, then each fractionation was performed according to protocol 1 described in the kit. Mitochondrial fractions were added to 50 µL of the same buffer, incubated on ice for 30 min, and vortexed. After being frozen at −80 °C for 30 min, they were incubated on ice for 30 min and vortexed. They were then frozen again at −80 °C for 30 min, added to 5 µL of 10% triton X (Wako Pure Chemicals, Osaka, Japan), and incubated on ice for 30 min. After centrifuging at 20,000× *g* for 10 min at 4 °C, the supernatant was used for TST and MST activity assays and western blot analyses for both enzymes.

### 2.3. TST and MST Activity Assays in Mitochondrial Fractions

MST activity catalyzes the trans-sulfuration from 3-mercaptopyruvate to 2-mercaptoethanol and TST activity from thiosulfate to cyanide. The activities of both enzymes were measured for each mitochondrial fraction obtained according to the method described above. MST and TST activities were measured by a modification of the methods of Vachek and Wood [29] and Sörbo [30], respectively, as described previously [17]. One unit of MST activity was defined as 1 µmol of pyruvate formed per minute, and one unit of TST activity was defined as 1 µmol of thiocyanate formed per minute.

### 2.4. Preparation of mRNA, cDNA Synthesis, and Real-Time Quantitative PCR Analysis for TST

Mouse livers were excised from littermate wild-type and KO mice, which were sacrificed by the method described above. Each mRNA was isolated using an mRNA Isolation Kit (Roche, Basel, Switzerland). The mRNA concentrations were measured using a NanoDrop Lite (Thermo Fisher Scientific). The cDNA samples were synthesized using a QuantiTect Reverse Transcription kit (QIAGEN, Venio, The Netherlands): Each mixture contained 1 μg of each RNA template, 2 μL of gDNA Wipeout Buffer, and RNase-free water for a final volume of 14 μL. Synthetic reactions were performed at 42 °C for 2 min. Further reactions were performed on the obtained 14-μL mixture, 1 μL of Quantiscript Reverse Transcriptase (RT), 4 μL of Quantiscript RT buffer, and 14 μL of RT Primer Mix at 42 °C for 15 min, and then 95 °C for 3 min.

Mouse TST primers were designed based on the GenBank nucleotide database [31] of mouse TST as follows: F (forward) primer, GGAGCCCGGATATAGTAGGACTAGA; and R (reverse) primer, TTCGTCAGGAAGTCCATGAA. Mouse glyceraldehyde 3-phosphate dehydrogenase primer (GAPDH, QuantiTect Primer Assay, QIAGEN) and mouse β-actin (QuantiTect Primer Assay, QIAGEN) were used as controls. The SYBR Green real-time quantitative PCR assays were performed in three different PCR systems using a QuantiTect SYBR Green PCR kit (QIAGEN; triplicates). The mixture contained 1 μL of DNA template (50 ng), 1.5 μL of each F and R primer (10 ng), and 25 μL of 2 × SYBR Green PCR Master Mix for a final volume of 50 μL. The PCRs were performed using an Applied Biosystems 7500 Real-Time PCR System (Thermo Fisher Scientific K.K., Tokyo, Japan). The following thermal cycling conditions were used: initial denaturation at 50 °C for 2 min and 95 °C for 15 min; followed by 45 cycles at 94 °C for 15 sec, 53 °C for 30 sec, and 72 °C for 35 sec; and by one cycle at 95 °C for 15 sec, 60 °C for 1 min, and 95 °C for 15 sec.

### 2.5. Western Blot Analysis of Mitochondrial Fractions for TST and MST

Mitochondrial fractions (wild-type #1, 2.7 mg/mL; KO #1, 3.2 mg/mL) were used for western blot analysis—each 30-mg sample was loaded into a 10% sodium dodecyl sulfate polyacrylamide gel electrophoresis (SDS-PAGE, 13.5 cm × 15 cm) chamber with a stacking gel. Each sample was added to 2% SDS, 100 mM Dithiothreitol (DTT), 0.1% bromophenol blue, and 10% glycerol in 50 mM Tris HCl buffer (pH 6.7; total volume, 22 μL), and then heated at 100 °C for 10 min. All reagents were purchased from Wako Pure Chemicals Industries, Ltd. (Osaka, Japan). Separated proteins were transferred to Immuno-Blot polyvinylidene difluoride (PVDF) (BIO-RAD.com, Tokyo, Japan) with an electrotransfer apparatus (Nippon Eido Corp., Tokyo, Japan).

Anti-rat MST rabbit polyclonal antibody was prepared and partially purified as described previously [1], and showed cross-reactivity with MST of other species [3] and also TST [3]. In this experiment, although the titer of the antibody for MST was present at more than 70 times the amount of that of TST [3], the anti-MST antibody was not treated with rat recombinant TST to reduce the cross-reacting with TST. On the other hand, anti-rat TST rabbit polyclonal antibody was newly produced using recombinant TST [1], and partial purification was performed using ammonium sulfate.

Western blot analyses with anti-MST and anti-TST polyclonal antibodies (1:1500 and 1:500, respectively) were performed. Alkaline phosphatase-conjugated goat anti-rabbit IgG (1:1500, Jackson ImmunoResearch Laboratories, Inc., West Grove, PA, USA) was used as a secondary antibody. A 5-Bromo-4-chloro-3-indolyl-phosphate/nitro blue tetrazolium (BCIP/NBT) Color Development Substrate (Promega, Madison, WI, USA) was used for staining alkaline phosphatase-conjugated probes.

### 2.6. Protein Determination

Protein concentrations were determined using a Coomassie protein assay kit (Pierce Biotechnology, Inc., Rockford, IL, USA) with crystalline bovine serum albumin (BSA, MP Biochemicals, Irvine, CA, USA) as the standard.

### 2.7. Statistical Analysis

The significance of difference between values was estimated with the Student’s *t*-test, and *p* values less than 0.05 were deemed significant. All results have been rounded to no more than three significant figures.

## 3. Results

### 3.1. TST and MST Activity Assays in Mitochondrial Fractions

**Figure 2:**
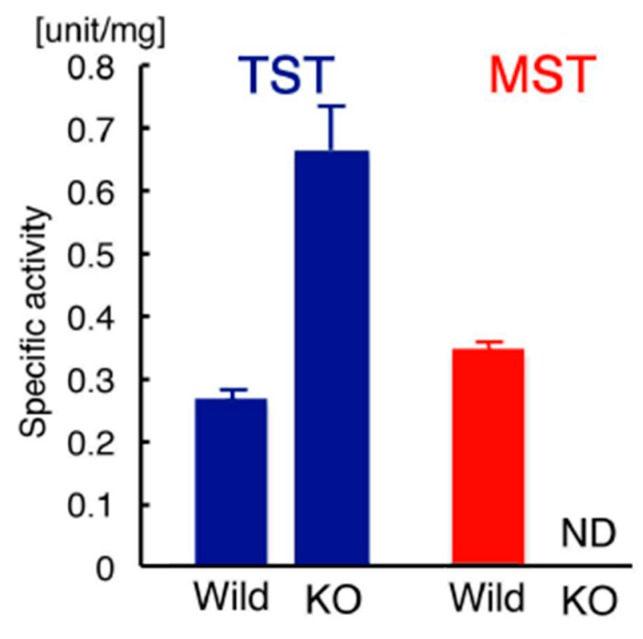
TST and MST activity assays in mitochondrial fractions. TST activity in KO mice was approximately 2.5 times more than the activity in wild-type mice (*p* = 8.99 × 10^−5^, *n* = 3). MST activity was not detected in KO mice. KO: MST-knockout mice; ND: not detected; Wild: wild-type mice.

TST activities in liver mitochondrial fractions of wild-type and KO mice (triplicate) were 2.65 ± 0.0331 × 10^−1^ and 6.61 ± 0.563 × 10^−1^ unit/mg, respectively (*p* = 8.99 × 10^−6^; Figure 2). MST activity of wild-type mice was 3.44 ± 0.0415 × 10^−1^ unit/mg (MST activity was not detected in KO mice; Figure 2). This experiment revealed that TST activity in KO mice was significantly increased compared to that in wild-type mice. MST activity was detected only in wild-type mice.

### 3.2. Preparation of mRNA, cDNA Synthesis, and Real-Time Quantitative PCR Analysis for TST

Isolated mRNAs of wild-type and KO mice were measured to be 5.36 × 10^2^ ng/μL (*A*_260_/*A*_280_ = 2.02) and 6.11 × 10^2^ ng/μL (*A*_260_/*A*_280_ = 1.99), respectively. The cDNA synthesis yields for wild-type and KO mice were 1.13 × 10^3^ μg/μL (*A*_260_/*A*_280_ = 1.85) and 1.15 × 10^3^ μg/μL (*A*_260_/*A*_280_ = 1.85), respectively.

**Figure 3:**
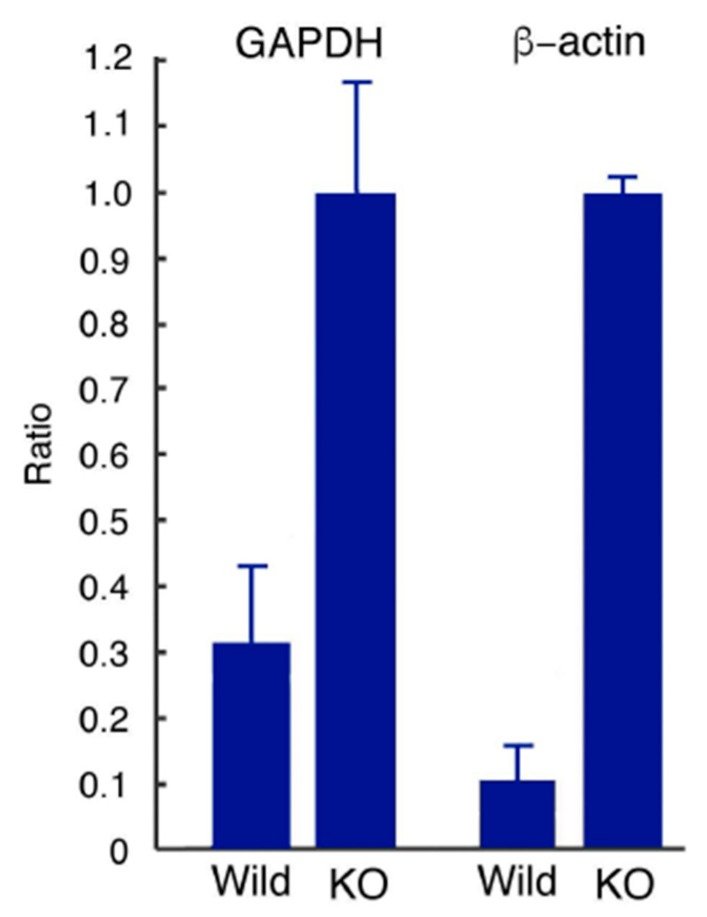
Real-time quantitative PCR analysis for TST mRNA. When GAPDH was used as an internal control gene, mRNA expression of TST in KO mice was approximately 3.3 times greater than expression in wild-type mice (*p* = 4.43 × 10^−3^, *n* = 3). On the other hand, when β-actin was used as the internal control gene, the ratio was approximately 9.1 (*p* = 6.69 × 10^−3^, *n* = 3).

Target genes for TST of wild-type and KO mice were measured as 4.42 ± 1.65 × 10^1^ and 1.03 ± 0.158 × 10^2^ bp, respectively (triplicate; *p* = 1.15 × 10^−2^). Internal control genes for glyceraldehyde-3-phosphate dehydrogenase (GAPDH) were 1.82 ± 0.119 × 10^2^ and 1.28 ± 0.150 × 10^2^ bp, respectively. Corrected values of TST mRNAs GAPDH (each target gene value/mean GAPDH gene value) for wild-type and KO mice were 2.43 ± 0.911 × 10^−1^ and 8.01 ± 1.23 × 10^−1^, respectively. The results of the revaluation were 3.03 ± 1.14 × 10^−1^ and 1.00 ± 0.154, respectively (Figure 3). On the other hand, control genes for β-actin were 5.04 ± 0.340 × 10^2^ and 1.29 ± 0.186 × 10^2^ bp, respectively. Corrected values of TST mRNAs (each target gene value/mean β-actin gene value) for wild-type and KO mouse were 8.75 ± 3.28 × 10^−2^ and 7.94 ± 0.122 × 10^−1^, respectively. The results of the revaluation were 1.10 ± 0.413 × 10^−1^ and 1.00 ± 0.0153, respectively (Figure 3). In conclusion, mRNA expression of TST in KO mice was significantly greater than in wild-type mice. This result is consistent with the results of our measurements of MST and TST activities.

### 3.3. Western Blot Analysis of Mitochondrial Fractions for TST and MST

**Figure 4:**
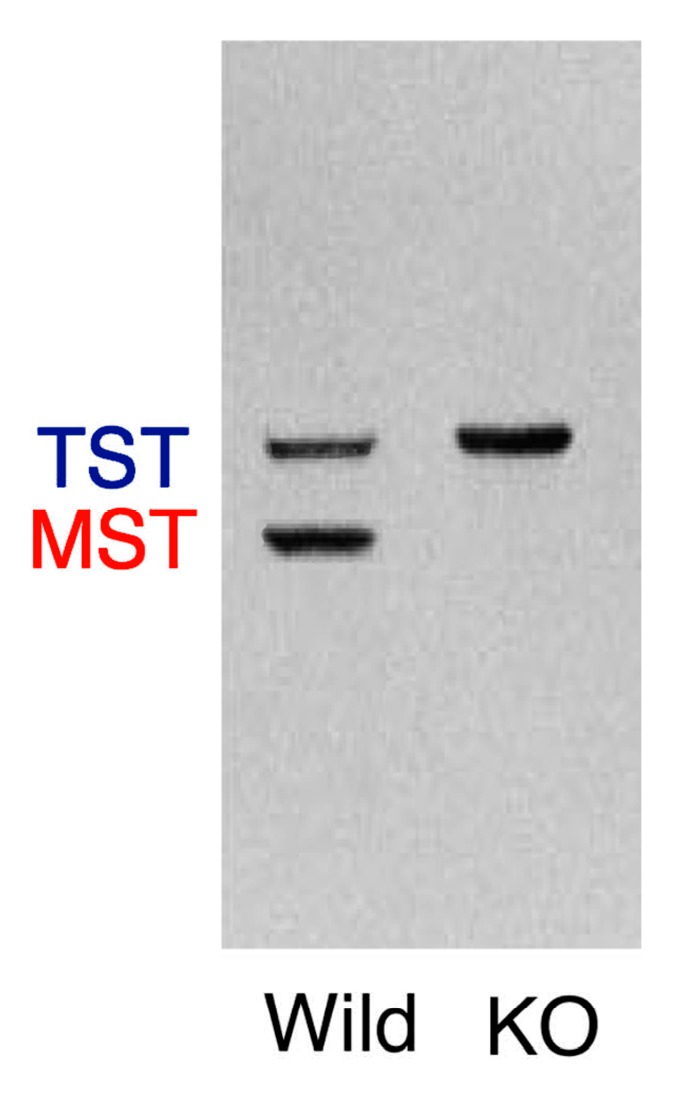
Western blot analysis of mitochondrial fractions for TST and MST. The band representing TST molecules from KO mice is more prominent than that of wild-type mice in the mitochondrial fractions.

Previous western blot analysis without blocking for TST revealed that the band representing TST molecules was more intense than the band representing MST, and their molecular masses were 33,180 and 32,808, respectively [2,3]. Anti-MST polyclonal antibody cross-reacted with TST [3]. In the present study, 30 mg samples of mitochondrial fraction proteins from wild-type and KO mice were loaded in gels. The band representing MST molecules was not detected in KO mice (Figure 4). On the other hand, the band representing TST molecules of KO mice was more prominent than that of wild-type mice (Figure 4), which is consistent with our measurement of the enzyme activities.

## 4. Discussion

**Figure 5:**
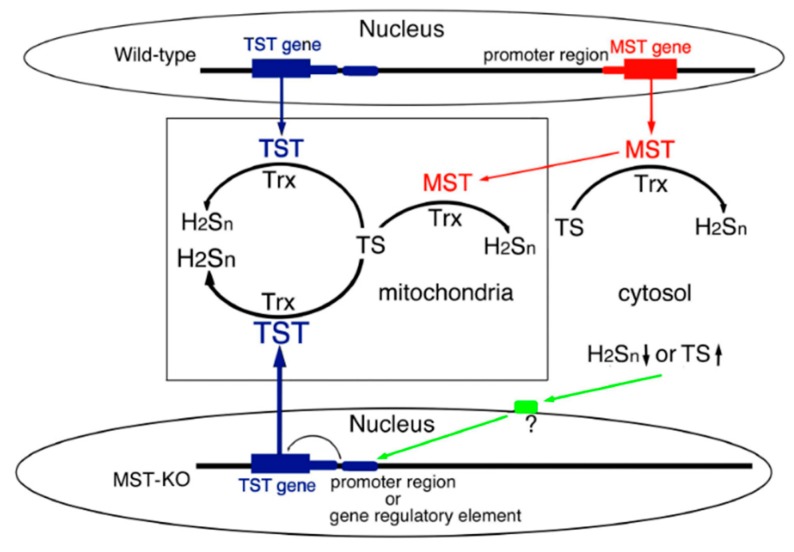
Proposed overexpression mechanism of TST. Decrease in cytosolic H_2_S_n_ may directly or indirectly affect the promoter region or the cis-regulatory element between TST and MST structural genes. Then it may facilitate transcription of TST gene, resulting in overexpression of TST. Trx: thioredoxin; TS: thiosulfate; H_2_S_n_: polysulfides. ?: unspecified factors.

TST and MST activity assays and western blot analysis of mitochondrial fractionation revealed that TST was overexpressed in MST-KO mice. Moreover, real-time quantitative PCR analysis supported this result. TST overexpression is, therefore, most likely compensating for the effect of the MST defect. TST and MST genes are aligned on both sides (NC_005106.4) (Figure 1 and Figure 5) and transcribed bidirectionally. When we first tried MST-KO mice production, we failed to obtain heterozygous KO mice [15]. The gene targeting was extended to 2714 bp from the initiation codon ATG of the promoter region, which in the germ line may cause an embryonic lethal phenotype. Therefore, a common regulatory region of transcription may be located in this area. In fact, the transcription of β-globin genes in erythroid cells is enhanced by the locus control region (LCR), which is localized in non-coding region at a long distance from the gene [32].

MST functions as an antioxidant [12,13,14,15,16,17], a producer of hydrogen sulfide and polysulfides [18,19,20,21,22], and as a possible producer of sulfur oxide [32]. After sulfuration of the catalytic site cysteine (Cys-S^−^ or Cys S-S^−^), reduction by thioredoxin produced hydrogen sulfide and polysulfide [20]. On the other hand, after oxidation of the sulfurated catalytic site cysteine to Cys-thiosulfenate (Cys-Sγ-SO^−^), Cys-thiosulfinate (Cys-Sγ-SO_2_^−^), and Cys-thiosulfonate (Cys-Sγ-SO_3_^−^), reduction of these molecules by thioredoxin was proposed to produce sulfur dioxides [33]. Hydrogen sulfide activates N-methyl-D-aspartic acid receptors via reduction of cysteine disulfide bonds in a ligand binding domain [23]; hydrogen sulfide directly acts on a functional protein in this case. Hydrogen sulfide also facilitates transcriptional factor, nuclear factor (NF)-κB via sulfuration [34]. Polysulfides facilitate the nuclear translocation of transcriptional factor NF-E2 related factor 2 (Nrf2) via sulfuration of Kelch-like erythroid-derived central nervous system homology factor associated protein 1 (Keap 1) [35]; polysulfides act on a transcriptional protein in this case.

On the other hand, decrease in hydrogen sulfide, polysulfides, and sulfur oxide or increase in thiosulfate could directly or indirectly regulate a functional protein; TST, a family enzyme of MST could be up-regulated (Figure 4), however, the transcription mechanism, *cis*-element of TST gene has not been clarified. Further, TST and MST are family enzymes and both enzymes catalyze the same substrates, although *K*_m_ and *K*_cat_ are different [1,2]. Thiosulfate, a substrate for both TST and MST [1,2], was accumulated in MST-KO mice (unpublished data), which may induce overexpression of TST (Figure 5).

## 5. Conclusions

The ratio of TST activity in MST-KO to wild-type mice was approximately 2.5. Real-time quantitative polymerase chain reaction analysis revealed the ratio was approximately 3.3. These facts led us to conclude that TST was overexpressed in the KO mice. Double (MST and TST)-KO mice, therefore, should be produced to investigate the pathophysiology of defects in H_2_S and H_2_S_n_ in mitochondria.
